# Multiparity Reduces Urethral and Vaginal Pressures Following the Bulboglandularis Muscle Stimulation in Rabbits

**DOI:** 10.1007/s43032-023-01263-3

**Published:** 2023-06-05

**Authors:** Cecilia Hernández-Bonilla, Diego Zacapa, René Zempoalteca, Dora Luz Corona-Quintanilla, Francisco Castelán, Margarita Martínez-Gómez

**Affiliations:** 1 Doctorado en Ciencias Biológicas, Universidad Autónoma de Tlaxcala, Tlaxcala, México; 2 Centro Tlaxcala de Biología de la Conducta, Universidad Autónoma de Tlaxcala, Tlaxcala, México; 3 Instituto de Investigaciones Biomédicas, Departamento de Biología Celular y Fisiología, Unidad Foránea Tlaxcala, Universidad Nacional Autónoma de México, Tlaxcala, México

**Keywords:** Parity, Pelvic floor, Striated muscles, Urogenital tract

## Abstract

Unlike male mammals showing a well-delimited external urethral sphincter, female mammals have urogenital sphincters shaped by muscles like the urethrovaginal sphincter. Childbirth-related injuries affect morphometry and function of urogenital sphincters in women, which frequently underlies pelvic floor disorders, including stress urinary incontinence and pelvic organ prolapse. The bulboglandularis muscle (Bgm) seems to shape a urogenital sphincter in rabbits. We determined herein the effect of multiparity on urethral and vaginal pressures generated by the Bgm stimulation in age-matched nulliparous and multiparous chinchilla-breed rabbits to stimulate the Bgm with trains of ascending frequencies (from 1 to 100 Hz; 4 s duration each). Subsequently, the Bgm was excised, measured in width, and weighed. Significant differences (*P* ≤ 0.05) were determined with Mann-Whitney *U* or Student *t*-tests or repeated measures two-way ANOVA followed by Tukey tests. Spearman’s partial coefficients were calculated to investigate the correlation between the highest pressure (urethral or vaginal) and the Bgm width. Multiparity reduced the weight and the width in the Bgm origin and medial regions. Urethral and vaginal pressures increased in response to the electrical stimulation of Bgm with frequencies from 20 to 100 Hz. Multiparas showed significant reductions in both types of pressures. We detected a strong correlation (conditioned by multiparity) between the medial Bgm width and the highest vaginal pressure. Our present findings demonstrate that multiparity impairs the function of Bgm, resulting in diminished urethral and vaginal pressures. Furthermore, the significant narrowness of the Bgm was correlated with the vaginal pressure recorded.

## Introduction

The striated muscle components of sphincteric muscle complexes, such as the urethral sphincter, urethral compressor muscle, and urethrovaginal sphincter (UVS), play an essential role in the mechanism of female urinary continence [1–3]. The UVS begins as a small tendon that is attached to the ischio-pubic ramus laterally at a point near the anterior border of the ischial tuberosity or on a plane at the posterior border of the vagina [1–3]. The UVS contraction compresses the urethra and vagina being responsible for ~ 41% of the urethral closure pressure [4, 5]. Childbirth-related levator ani and sphincter complex injuries drive to the onset of stress urinary incontinence (SUI) [4, 5]. Women suffering SUI at postpartum display low urethral closure pressures [4, 5] and a small cross-sectional area of the sphincter complex [6].

Models approaching urethral dysfunctions in female mammals have coped with disagreements in nomenclature and anatomical organization of muscles contributing to the urethral sphincter [1, 7]. The female rabbit is a well-suited species to study the lower urogenital tract (LUT) physiology, including pelvic floor muscles (PFM) [8–13]. The synchronized activity of muscles like bulbospongiosus (Bsm) and pubococcygeus (Pcm) contributes differently to urine continence and voiding [10, 14]. Besides internal urethral sphincters inferred by histological findings [15], the urethral closure in rabbits depends on skeletal muscles assisting or shaping sphincteric complexes [14, 16, 17]. We have proposed elsewhere that the bulboglandularis muscle (Bgm), composed of circular myofibers enveloping the vestibular glands, distal urethra, and pelvic vagina in rabbits, resembles the UVS in women [1, 4, 7, 12]. During the storage phase, the Bgm shows bursts of tonic activity that gradually decrease until turning off at the onset of the voiding phase of micturition [17].

Bgm has also been reported for the female cat [18] that have a LUT that shares a closer anatomy with that of female rabbits, including the presence of a urogenital sinus designated as vestibulum [11] and observed at the level of the pelvic (or medial) vagina [9]. Nevertheless, the Bgm has also been reported for female mammals exhibiting two separate urinary and reproductive ducts, like cows [19]. By contrast, the muscle urethralis in female dogs shows striking anatomical and histological similarities regarding the Bgm in rabbits [17, 20]. Understanding the functional organization of muscles shaping sphincteric complexes can lead to a better comprehension of female urethral physiology.

Multiparity (four successive deliveries) affects urodynamics and modifies the activation of Bsm and Pcm, as well as bladder and urethral pressures in young rabbits [8, 21], and impairs the contractile force of the Bsm, ischicavernosus (Ism), and Pcm matching alterations in the vaginal pressure [22]. Moreover, the histology of the caudal urethra and pelvic vagina is disorganized [15]. In aged multiparous rabbits, the EUS is thinner and vaginal, and urethral pressure is reduced [23], and the urethral pressure recorded at micturition reflex is lower [21]. Overall, we hypothesized that multiparity impairs the function of Bgm, resulting in diminished urethral and vaginal pressures. Therefore, the present study aimed to determine urethral and vaginal pressures generated by the electrical stimulation of the Bgm in nulliparous and multiparous rabbits.

## Materials and Methods

### Animals

Twelve chinchilla-breed female rabbits (*Oryctolagus cuniculus*) were randomly allocated (by raffle) to nulliparous (N, *n* = 6) and multiparous (M, *n* = 6) groups. The rabbits were housed individually in stainless-steel cages and kept at 20–28 °C under artificial long-day conditions (L:D 16:8, lights on at 0600) and were provided daily with pellet food (Conejina, Purina) and had continuous access to water.

Multiparous rabbits had four consecutive and successive deliveries. Female rabbits had the first mating at 6 months of age; subsequent mating was allowed on the first day postpartum after deliveries 1–3, so they were pregnant and lactating for 20 days when pups were weaned. On the day of the fourth delivery, neonate pups were euthanized to avoid lactation and allow multiparous rabbits reaching a hormonal condition like nulliparas (virgin) as determined by the estradiol levels in serum on the postpartum day 20 [19]. Physiological recordings were done under anesthesia with urethane (1.5 g/kg, i.p.; Sigma-Aldrich, México) in multiparas on the postpartum day 20 and in age-matched nulliparas. Therefore, we evaluated the effects of multiparity on urethral and vaginal pressures. At the end of the following experimental procedures, the rabbits were euthanized with an overdose of urethane. No animals were excluded from the following experiments. The experiments were conducted at the Tlaxcala Unit of the Instituto de Investigaciones Biomédicas of the Universidad Nacional Autónoma de México (IIBo-UNAM). The Ethics Committee from the IIBo-UNAM approved all the experimental procedures.

### Bgm Dissection

**Fig. 1:**
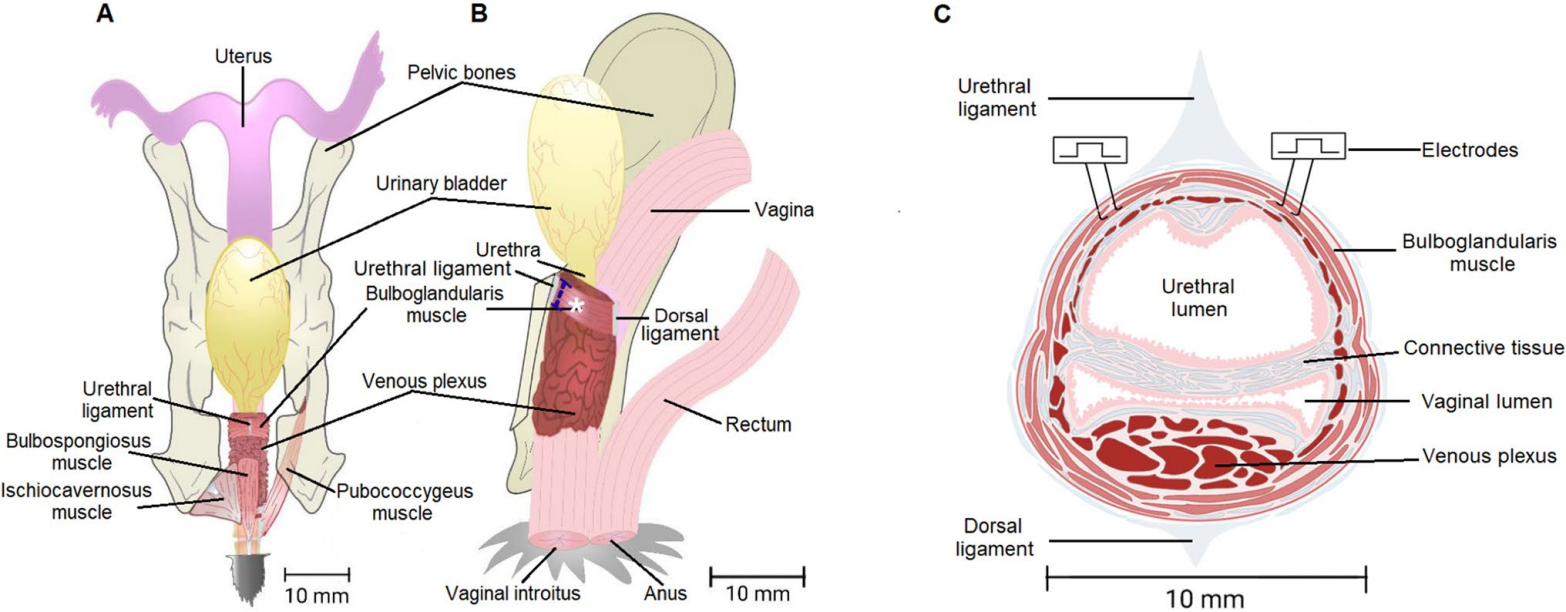
Schematics of lower urogenital tract and bulboglandularis muscle (Bgm) in rabbits. Ventral (**A**) and lateral (**B**) views depicting the Bgm runs from the urethral ligament (ventrally) to the dorsal ligament. The measurement of the width at the origin of the Bgm is indicated with a dotted line. The venous plexus covers much of the pelvic vagina and is conspicuous in the distal part of the bladder. *Asterisk* denotes the site in which the width of the medial region of the Bgm was measured. **C** At the level of the urogenital sinus, the Bgm is circularly oriented, encircling the venous plexus, pelvic vagina, and caudal urethra. Stimulating electrodes were placed at the medial region of the Bgm length

Rabbits (N, *n* = 6; M, *n* = 6) were anesthetized with urethane (1.5 g/kg, i.p.; Sigma-Aldrich, México) and fixed in a dorsal supine position to incise the skin and abdominal muscles. We carefully removed the pubic symphysis as it covers the urogenital tract widely. Under a surgical microscope (SMZ-2 T, Nikon, Japan), lateral cuts were made in the ischial arch and the distal part of the bladder. When the bone was separated, the Bsm and external obturator were disinserted to expose the internal obturator muscles. Subsequently, the origin and insertion of the internal obturator muscle were cut to observe the Bgm. After, a thin layer of striated fibers of Bgm was located ventral and surrounded the pelvic vagina, which is covered by the pelvic plexus. The fibers of Bgm were attached by the named urethral ligament (Fig. 1A, B). Next, we carefully cut the internal obturator myofibers that joined the urethral ligament to avoid transecting some external fibers of Bgm. Finally, the Bgm width was measured at its origin and its medial and proceeded with measures of urethral and vaginal pressures during the electrical stimulation of the Bgm.

### Urethral and Vaginal Pressure Generated by the Bgm

Urethral and vaginal pressures were recorded as reported elsewhere [17, 22]. The bladder was exposed through a midline incision in the abdomen, and mild pressure was manually applied to expel the urine. Subsequently, a catheter (1.6-mm O.D. and 1.2-mm I.D.) was introduced into the urogenital tract through the bladder apex and pulled across the urethra and vagina until it appeared out of the vaginal opening. Next, the catheter was tied to a balloon with a cotton thread (8 cm in length) and was introduced inside the urethral tract (5.5–6 cm), specifically at the level of the caudal urethra. Next, the balloon was moved toward the pelvic vagina (5 cm) to record the vaginal pressure sequentially in both regions. The balloon was then filled with warm saline (39 °C; 0.9% NaCl) until the urethral and vaginal diameter was reached. Next, the catheter was connected to a P23BC pressure transducer (Statham, HatoRey, Puerto Rico) to record pressure variations and connected to a Grass 7DA D.C. amplifier. The balloon was pressed manually until a significant increase in pressure was observed in the recording system to confirm the correct position of the balloon in the caudal urethra or pelvic vagina. Data were displayed and stored in the program PolyView Recorder 2.5 (Grass) for analysis.

To determine the contribution of the Bgm in the generation of pressure on the urethra and pelvic vagina, we provoked the muscular contraction with constant voltage square pulses and stimulation trains of ascending frequency. Bipolar silver electrodes (0.1 mm diameter) were placed bilaterally in the medial region of Bgm (Fig. 1C). Firstly, single square voltage pulses (1 Hz, 0.5 ms duration) were applied through a stimulus isolation unit SIU5 (Grass Instruments, Quincy, MA, USA) activated by an S48 stimulator (Grass Instruments) to determine the stimulation threshold of the muscle. Stimulation amplitude was gradually increased until observing a minimal muscle contraction and a slight deflection in the recorded pressure; once the threshold stimulus strength (STh; volts, V) was determined, we multiplied it by two, three, and four (4 × STh) ﻿to stimulate the muscle and reach its maximal response. Secondly, an ordered series of constant voltage stimulation trains were sequentially applied to induce a tetanic contraction and the maximum pressure; the electrical stimulus was applied with frequencies of 1, 4, 10, 20, 50, and 100 Hz (4 × STh, 4 s duration each). Each stimulation frequency was applied in triplicate and spaced by rest periods of 8 min to avoid muscle fatigue. The average values of both urethral and vaginal pressures were measured in all the rabbits from the N and M groups; the resulting measures were expressed in mmHg. After the physiological records, the Bgm was excised and weighed.

### Statistical Analysis

Data are median (minimum to maximum range) or mean ± standard error of the mean (S.E.M.) (*n* = 6 per group). The normality of data was assessed with Kolmogorov–Smirnov tests. We used two-tail unpaired Mann–Whitney *U* or Student *t*-tests to determine significant differences (*P* < 0.05) regarding the age, body, and Bgm masses, Bgm width, and STh. We used two-way ANOVA tests to determine significant differences to inspect if Bgm stimulation frequencies and multiparity affected the urethral and vaginal pressures; Tukey post hoc tests were carried out for pairwise comparisons. The latter statistical analyses were done with the Prism program version 6 (GraphPad Software, San Diego, California). We used the R-based JASP software (v. 0.16.4) [24] to calculate Spearman’s *rho* partial coefficients to determine the correlation between pressures and Bgm width, as conditioned by the median number of delivered pups.

## Results

Table 1 presents zoometric variables of nulliparous and multiparous rabbits including the Bgm morphometry. Of notice, the Bgm wet mass was lower in multiparas than in nulliparas, but the normalized Bgm mass did not vary significantly. The width at the proximal and medial regions of the Bsm was reduced in multiparous rabbits.

**Table 1:** Zoometric variables of nulliparous and multiparous rabbits

	Nulliparas (*n* = 6)	Multiparas (*n* = 6)	Test statistic	*P*-value
Age,* months*	11 (10–12)	12 (11–12)	*U* = 7	0.1
Body mass,* kg*	3.9 ± 0.1	3.6 ± 0.1	*t* = 1.8	0.1
Pups per delivery	NA	8.7 (6–10)	NA	NA
Pup body mass,* g*	NA	52.5 ± 2.6	NA	NA
Bgm wet mass,* mg*	292 ± 33	190 ± 24	*t* = 2.5	**0.0307**
Bgm normalized mass,* mg/kg body mass*	76 ± 11	54 ± 8	*t* = 1.6	0.1
Bgm width				
*at origin, cm* *at middle, cm*	1.1 ± 0.07 1.8 ± 0.2	0.8 ± 0.03 1.02 ± 0.03	*t* = 2.7 *t* = 3.8	**0.0231** **0.0034**

Data are medians (minimum–maximum) or means ± S.E.M. Pair group comparisons were made with Mann–Whitney *U* (*U*) or Student-*t* (*t*) tests. *NA*, not applicable. Significant *P* values are indicated in *bold*

### Urethral Pressure During Electrical Stimulation of the Bgm

**Fig. 2:**
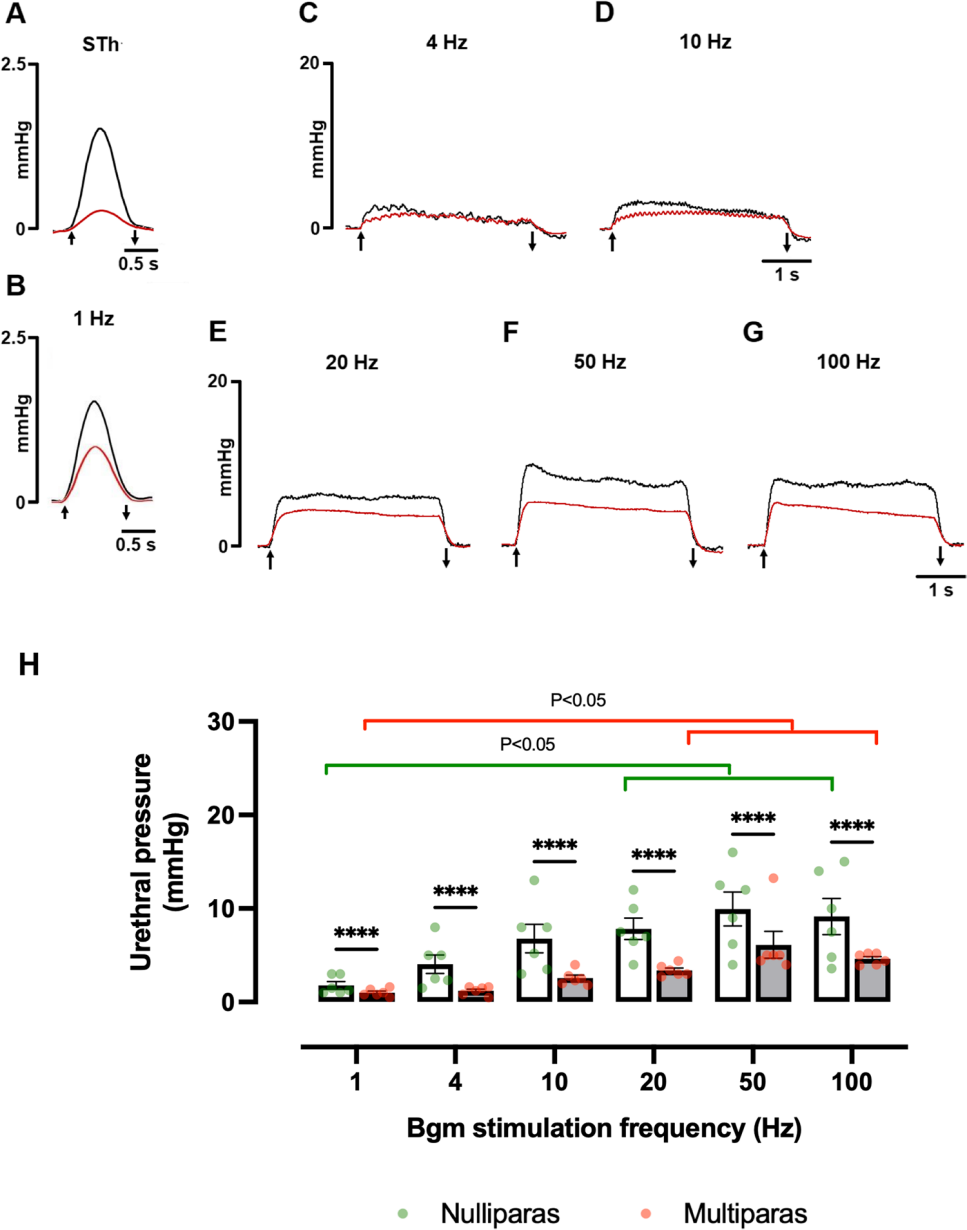
Multiparity affects the urethral pressure produced by stimulating the Bgm in rabbits. Representative recordings of the urethral pressure obtained during the application of the stimulus threshold (**A**) and stimulation trains of ascending frequencies (**B**–**G**) to the Bgm of nulliparous (*black traces*) and multiparous rabbits (*red traces*). Stimulus duration: 1 s (**A**, **B**), 4 s (**C**–**G**). A*rrows* indicate the stimulation period. **H** Data are means ± SEM, *n* = 6 per group. Significant differences (*P* < 0.05) between groups were assessed with ordinary two-way ANOVA followed by Tukey tests. * *P* < 0.05; ** *P* < 0.01. *mmHg*, millimeters of mercury; *Hz*, Hertz; *s*, second

Figure 2A–G shows representative recordings of urethral pressure during the electrical stimulus threshold (STh) and ascending frequency trains applied to the Bgm. The STh that triggered the minimum urethral pressure did not vary between nulliparas and multiparas (0.85 ± 0.11 vs. 0.63 ± 0.07 V; *t* = 1.6, *P* = 0.147). In contrast, multiparity reduced the urethral pressure in response to the STh (0.36 ± 0.04 vs. 1.27 ± 0.09 mmHg; *t* = 9.2, *P* < 0.0001).

The STh was multiplied by four (4 × STh) to obtain maximal muscle responses (Fig. 2 B–G). The urethral pressure changed following the Bgm electrostimulation at 1, 4, and 10 Hz (Fig. 2B–D) and acquired a characteristic tetanus shape by stimulating at 20, 50, and 100 Hz (Fig. 2E–G). Both the Bgm stimulation at different frequencies and multiparity significantly influenced the urethral pressure (StimFreq: F_(5,65)_ = 10.9, *P* < 0.0001; multiparity: F_(1,65)_ = 31, *P* < 0.0001). Indeed, we detected a significant increase in the urethral pressures recorded to stimulate at frequencies higher than 10 Hz in nulliparas (1 vs. 20 Hz, *P* = 0.0113; 1 vs. 50 Hz, *P* < 0.0001; and 1 vs. 100 Hz, *P* = 0.0002) and multiparas (1 vs. 20 Hz, *P* = 0.0113; 1 vs. 50 Hz, *P* < 0.0001; and 1 vs. 100 Hz, *P* = 0.0002) (Fig. 2H). Post hoc tests also showed that multiparity decreased significantly the urethral pressures recorded after stimulating the Bgm with each frequency (Fig. 2H).

**Fig. 3:**
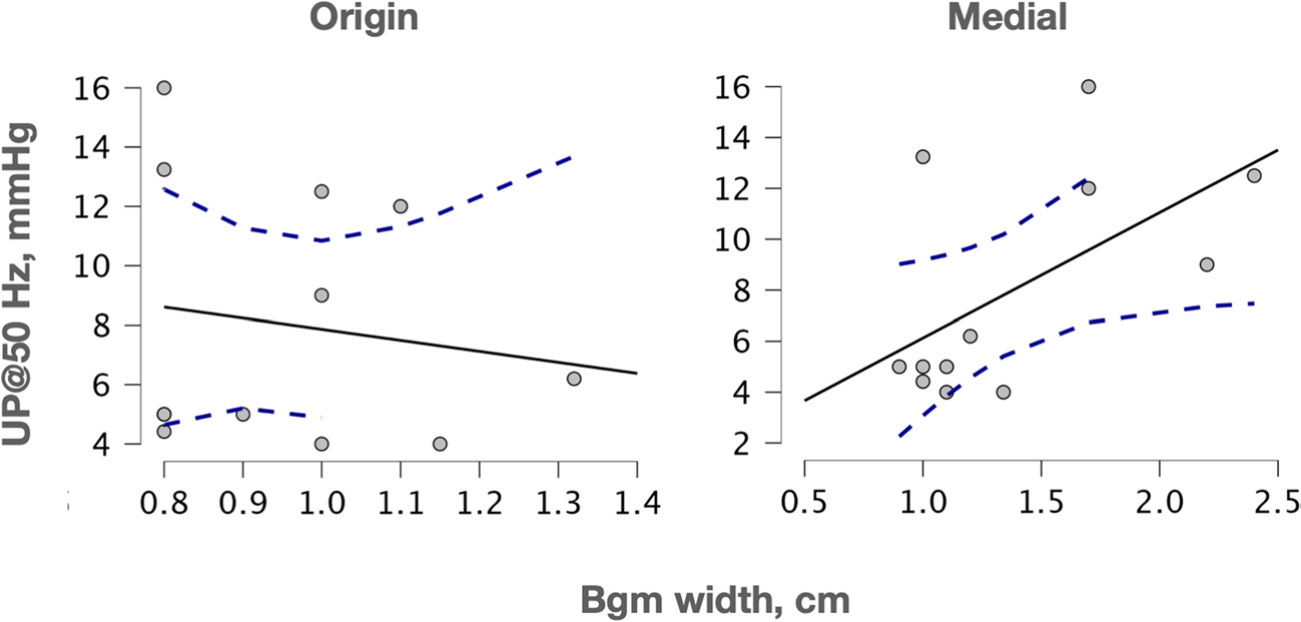
Correlation between the Bgm with at the origin or medial regions with the urethral pressures raised during the Bgm stimulation at 50 Hz (UP@50Hz), as conditioned by the number of delivered pups. Dashed lines indicated the 95% confidence intervals. Hz, Hertz; mmHg, milimeters of mercury

We reasoned that a narrower Bgm could correlate with the low urogenital pressures recorded in multiparous rabbits. Therefore, we determined Spearman’s *rho* partial coefficients between the urethral pressure at 50 Hz (considered as the maximum) and the Bgm width at its origin, close to the urethral ligament, or at its medial region, conditioning such analyses by the (median) number of pups delivered, assigning a 0-value to nulliparas. We observed thus a significant negative correlation between the maximal urethral pressure and the Bgm width at its origin (Spearman’s *rho* =  − 0.68, *P* = 0.022), but not with its medial region (Spearman’s *rho* = 0.23, *P* = 0.491; Fig. 3). The width of both Bgm regions was not significantly correlated (Spearman’s *rho* =  − 0.24, *P* = 0.469).

### Vaginal Pressure During Electrical Stimulation of the Bgm

**Fig. 4:**
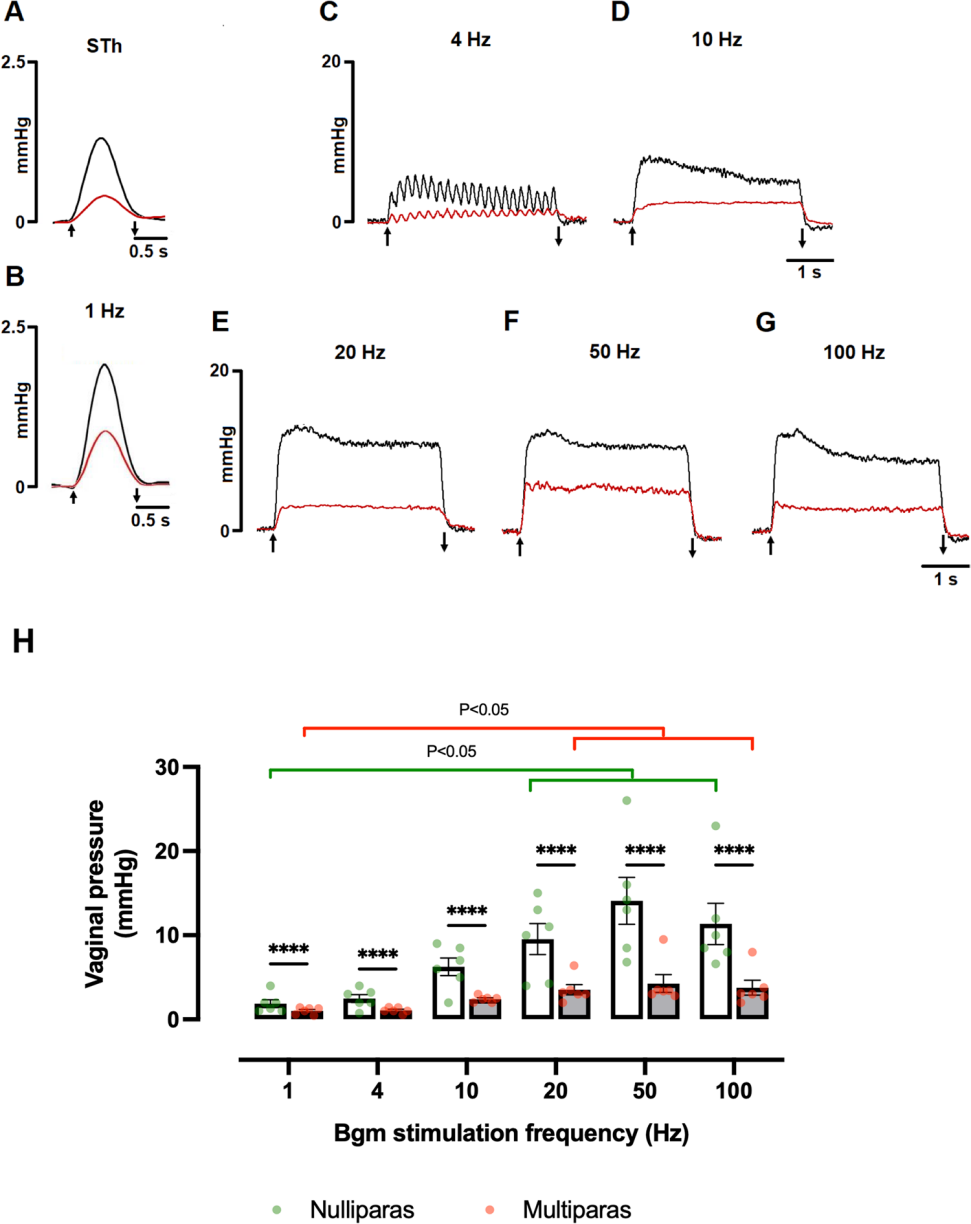
Multiparity affects the vaginal pressure produced by stimulating the Bgm in rabbits. Representative recordings of the vaginal pressure obtained during the application of the stimulus threshold (**A**) and stimulation trains of ascending frequencies (**B**–**G**) to the Bgm of nulliparous (*black traces*) and multiparous rabbits (*red traces*). Stimulus duration: 1 s (**A**, **B**), 4 s (**C**–**G**). A*rrows* indicate the stimulation period. **H** Data are means ± SEM, *n* = 6 per group. Significant differences (*P* < 0.05) between groups were assessed with two-way ANOVA followed by Tukey tests. * *P* < 0.05; ** *P* < 0.01. *mmHg*, millimeters of mercury; *Hz*, hertz; *s*, second

The STh of the Bgm at the pelvic vagina significantly decreased in multiparas (0.70 ± 0.06 vs. 1.00 ± 0.02 V; *t* = 4.1, *P* = 0.002); the same was true for the matched vaginal pressure (0.53 ± 0.04 vs. 1.29 ± 0.06 mmHg; *t* = 9.9, *P* < 0.0001; Fig. 4A). The vaginal pressure rose during the Bgm stimulation at 4–100 Hz and after returned to its baseline (Fig. 4B–G).

The vaginal pressure was significantly affected by the Bgm stimulation triggered by the application of different frequencies (F_(5,65)_ = 9.5, *P* < 0.0001) and multiparity (F_(1,65)_ = 35, *P* < 0.0001). As compared with pressures resulting to stimulate at 1 Hz (Fig. 4H), we recorded higher pressures during the stimulation of the Bgm at 20, 50, and 100 Hz for nulliparous (1 vs. 20 Hz, *P* = 0.0355; 1 vs. 50 Hz, *P* < 0.0001; and 1 vs. 100 Hz, *P* = 0.004) and multiparous (1 vs. 20 Hz, *P* = 0.0355; 1 vs. 50 Hz, *P* < 0.0001; and 1 vs. 100 Hz, *P* = 0.004) rabbits. Multiparity decreased significantly the vaginal pressures recorded during the stimulation of the Bgm with each of the applied frequencies (Fig. 4H).

**Fig. 5:**
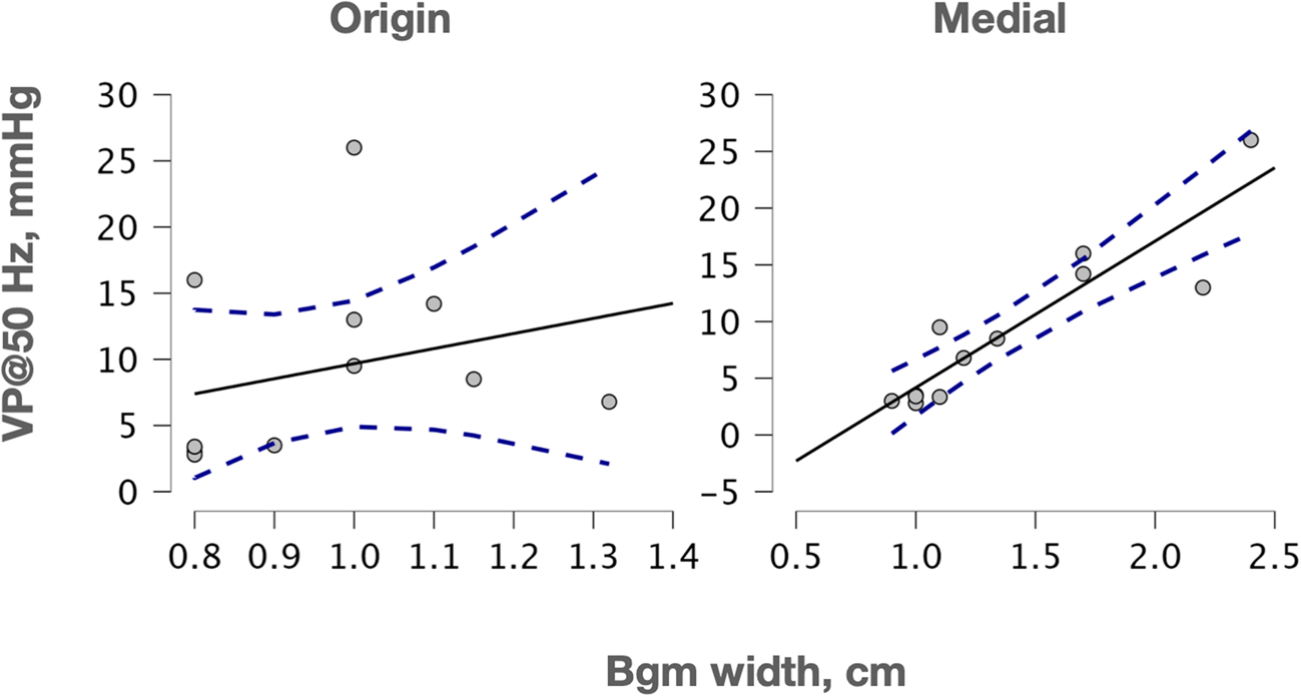
Correlations between the Bgm with at the origin or medial regions with the vaginal pressures raised during the Bgm stimulation at 50 Hz (VP@50Hz), as conditioned by the number of delivered pups. Dashed lines indicated the 95% confidence intervals. Hz, Hertz; mmHg, milimeters of mercury

As approached for the urethral pressure, we determined Spearman’s *rho* partial coefficients between the vaginal pressure at 50 Hz and the Bgm width at its origin or medial region, conditioning the analysis by the number of pups delivered (Fig. 5). We observed a significant correlation between the vaginal pressure and the Bgm width at its medial region (Spearman’s *rho* = 0.66, *P* = 0.028). No significant correlation was found between the maximum vaginal pressure and the Bgm width at its origin (Spearman’s *rho* =  − 0.25, *P* = 0.451).

## Discussion

Childbirth damages the urethral rhabdosphincter and levator ani as supported by ultrasound- or MRI-imaging regarding the length, thickness, volume, and other parameters [5, 25, 26]. Indeed, other pelvic tissues like the venous plexus, the compressor urethral and the UVS collaborate in elevating the urethral pressure [2]. We have previously proposed that the Bgm could serve as a UVS able to elevate urethral and vaginal pressure in rabbits [17]. Our findings herein demonstrate that multiparity leads to a narrower Bgm that generates low urethral and vaginal pressures during its stimulation. Furthermore, we found that only the vaginal pressure and the medial Bgm width correlate positively.

The Bgm width at the origin and medial regions was significantly reduced in multiparous rabbits. Such a finding agrees with data from multiparous rabbits regarding the morphometry of the Bsm, but not for that of the Pcm [27] neither for both muscles of primiparous rabbits [28]. Given the Bsm runs along the clitoral ligament at the ventral vagina, and the Bgm encircles the urogenital sinus, the vaginal distention because of four consecutive deliveries may be stretching out both the Bsm and Bgm. Such findings match significant affectation in urethral and vaginal pressures.

The contraction of PFM like the puborectalis and Bgm assist the elevation of urethral pressure in female rabbits [16, 17, 23, 29]. Our findings herein show that the highest urethral pressure was measured during the Bgm stimulation between 20 and 100 Hz for nulliparous and multiparous rabbits. Certainly, the pressures recorded after the stimulation with such, and even lower, frequencies were significantly reduced by multiparity. Present data agree with the decreased pressure to close the urethra reported for multiparous and aged rabbits in response of electrostimulation of the external urethral sphincter [29] and during the micturition reflex [21]. Likewise, the multiparity-associated reduction in type II myofibers and/or its conversion to type I reported for the external urethral sphincter that “encircles both the bladder neck and the distal end of the vagina,” of rabbits is also in line with findings herein [30]. In addition, the decrease in the number of striated muscle cells in old multiparous could contribute to decreased urethral pressure and cause an inadequate closure during the urinary continence [31]. Otherwise, the distal (caudal) urethra histology associated with multiparity, namely, the reduced content of collagen, blood vessels, and striated muscle, possibly involved in fibrosis and/or EUS atrophy, could explain the low urethral pressures recorded herein [15, 29]. Moreover, the reflex activation of other PFM during the micturition reflex (e.g., the Pcm, Bsm, and ischiocavernosus muscles) is also able to modulate the urethral function [21].

In a highly similar fashion to the case of the urethra, the vaginal pressure raised during the Bgm stimulation. Such an effect is well-related with data reported for the electrostimulation of puborectalis [29], ischiocavernosus, Bsm, and the Pcm at low frequencies [22, 23]. In comparison with nulliparas, we recorded lower vaginal pressures of multiparas resulting from the Bgm stimulation at all tested frequencies, which agrees to the responses reported during the Bsm and Ism stimulation [22]. Furthermore, present findings could be associated with significant reductions in the thickness of the epithelial layer and the blood vessel content in the pelvic vagina [15]. In ewes, multiparity, to a greater extent than primiparity, impairs vagina structure and function, impacting the vaginal wall thickness, muscular content, elastin fibers, and pressure [32]. Remarkably, alterations in the vagina from multiparous ewes are considered potential indicators of pelvic disorders like pelvic organ prolapse [32, 33].

Parous women are prone to develop pelvic floor dysfunctions through stretching pelvic floor nerves and muscles, impairing the contraction of muscles like the LA [34]. Indeed, reduced dimensions regarding urethral length and area, among others, are factors influencing low urethral pressures in women [35]. Furthermore, defects on morphometry of the levator ani in women suffering POP associate with a reduction in the vaginal closure force [5, 36]. In conditioning by the number of delivered pups, we observed a strong correlation between the medial Bgm width and the highest vaginal pressure, but not with the urethral one (both pressures measured at 50 Hz), which was unexpected given the stimulating electrodes were placed nearest to the latter than the former viscera (Fig. 1). Certainly, the medial Bgm width was reduced about two-fold as compared to the affectation of the width at the Bsm origin. Indeed, multiparity reduces the contractile force developed by the Bsm and Ism matching low vaginal pressures in rabbits, which may contribute synergistically to the low vaginal pressure [22]. Besides analyzing the contribution of vaginal smooth muscle as it contributes differentially to the genesis of vaginal pressure in women [37], further approaches should explore changes in the integrity of paravaginal tissue due to its lacking may explain the sharp reduction in the vaginal pressure of multiparous rabbits. In support of the latter proposal, ganglia morphometry of the pelvic plexus embedded on paravaginal tissue has been found to be altered in multiparous rabbits [38].

A main limitation of our study was that the frailty of the Bgm hindered measuring its contractile force in nulliparas and multiparas. However, the present findings strengthen the role of coordinated activation of the different PFM in the physiology of the LUT. Striated fibers of the Bgm surrounding the distal urethra and vagina of the rabbit resemble a urethrovaginal sphincter [17]. Such anatomical disposition can explain the contribution of the Bgm and other PFM, like the bulbospongiosus, pubococcygeus, and puborectalis, to the increase in vaginal pressure [16, 22]. Nevertheless, only the contraction of the pubococcygeus [22] and the Bgm (shown herein) elevate the pressure at the level of the pelvic vagina, which implies that the synchrony in reflex activation of the PFM should also be considered.

## Conclusion

The multiparity reduces the Bgm contraction to generate, mainly, the urethral pressure and, with other pelvic floor muscles, the vaginal pressures. The pressure reduction would produce an inadequate urethral closure during the urinary continence affecting the sphincter function.

## Data Availability

The data supporting this study’s findings are available from the corresponding author (MMG) upon reasonable request.
